# Supplementary material for: Complex‐centric proteome profiling by SEC‐SWATH‐MS
Source: Mol Syst Biol. 2019 Jan 14;15(1):e8438. doi: 10.15252/msb.20188438 (PMC6346213; doi:10.15252/msb.20188438)
Supplement: Supplementary file 8 — Dataset EV7 [file MSB-15-e8438-s008.zip › feature_plots_string/Q13601.pdf]

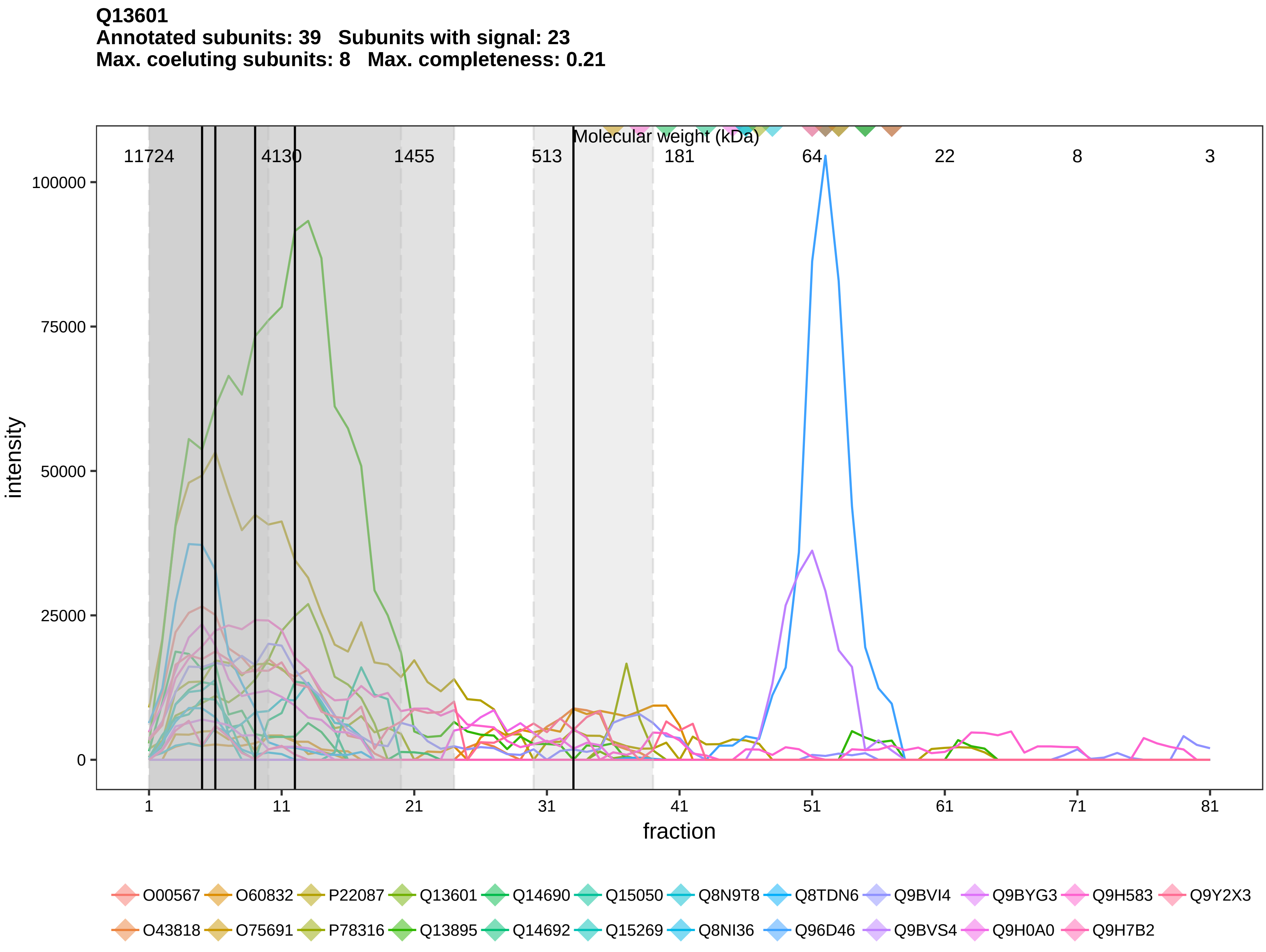
Q13601
Annotated subunits: 39 Subunits with signal: 23
Max. coeluting subunits: 8 Max. completeness: 0.21
Molecular weight (kDa)
11724
4130
1455
513
181
64
22
8
3
intensity
100000
75000
50000
25000
0
1
11
21
31
41
51
61
71
81
fraction
O00567
O60832
P22087
Q13601
Q14690
Q15050
Q8N9T8
Q8TDN6
Q9BVI4
Q9BYG3
Q9H583
Q9Y2X3
O43818
O75691
P78316
Q13895
Q14692
Q15269
Q8NI36
Q96D46
Q9BVS4
Q9H0A0
Q9H7B2
